# Age-Related Changes in Trabecular Meshwork Imaging

**DOI:** 10.1155/2013/295204

**Published:** 2013-09-19

**Authors:** Mark E. Gold, Seema Kansara, Kundandeep S. Nagi, Nicholas P. Bell, Lauren S. Blieden, Alice Z. Chuang, Laura A. Baker, Kimberly A. Mankiewicz, Robert M. Feldman

**Affiliations:** ^1^Ruiz Department of Ophthalmology and Visual Science, The University of Texas Medical School at Houston, 6431 Fannin Street, MSB 7.024, Houston, TX 77030, USA; ^2^Department of Ophthalmology, The University of Texas Health Science Center at San Antonio, 7703 Floyd Curl Drive, Mail Code 6230, San Antonio, TX 78229, USA; ^3^Robert Cizik Eye Clinic, 6400 Fannin Street, Suite 1800, Houston, TX 77030, USA

## Abstract

*Purpose*. To evaluate the normal aging effects on trabecular meshwork (TM) parameters using Fourier domain anterior segment optical coherence tomography (ASOCT) images. *Patients and Methods*. One eye from 45 participants with open angles was imaged. Two independent readers measured TM area, TM length, and area and length of the TM interface shadow from 3 age groups (18–40, 41–60, and 61–80). Measurements were compared using stepwise regression analysis. *Results*. The average TM parameters were 0.0487 (±0.0092) mm^2^ for TM area, 0.5502 (±0.1033) mm for TM length, 0.1623 (±0.341) mm^2^ for TM interface shadow area, and 0.7755 (±0.1574) mm for TM interface shadow length. Interobserver reproducibility coefficients ranged from 0.45 (TM length) to 0.82 (TM area). TM area and length were not correlated with age. While the TM interface shadow length did not correlate with age, the TM interface shadow area increased with age. Race, sex, intraocular pressure, and gonioscopy score were not correlated with any TM parameters. *Conclusion*. Although the TM measurements were not correlated with age, the TM interface shadow area increased with age. Further study is required to determine whether there is any relationship between the age-related ASOCT findings of the TM interface shadow area and physiologic function.

## 1. Introduction

The trabecular meshwork (TM) [1] is the primary drainage structure for aqueous humor and is intimately related to the pathophysiology of glaucoma. Because changes to the TM structure and function can have detrimental effects on the eye, understanding and preventing these potential consequences have been a source of interest for many decades. Beginning in the 1980s, decreasing cellularity of the TM, as seen in enucleated eyes, began to provide clues regarding the decreased aqueous outflow observed in aging patients [2]. Later that decade, McMenamin et al. demonstrated that the general configuration of the TM changes with age, from a “long wedge shape to a shorter, more rhomboidal form,” and also observed decreased cellularity and thickening of the TM [1]. Although the insight provided from fixed tissue histology studies is certainly valuable, cell death and processing artifacts may significantly alter measurements in unknown ways. 

Quantitative, *in vivo* data of the anterior segment became available with the creation of ultrasound biomicroscopy (UBM) and anterior segment optical coherence tomography (ASOCT). Many studies were performed to compare the accuracy and reproducibility of these 2 imaging modalities when measuring the anterior segment parameters [3–7]. As the imaging capability continues to improve, the TM [8] and its individual surrounding structures are now visible, including the scleral spur [9], Schlemm's canal [10–12], and Schwalbe's line [13]. In addition, new concepts have been characterized, including the TM interface shadow [11], as observed using ASOCT. However, there is currently no published evidence that this density-dependent shadow produced by the TM light reflections offers a new avenue to better understand the physiologic changes seen in TM tissue with age.

With the visible TM borders, reproducible quantification of the length and the size of TM was recently accomplished by Usui et al. [13]. The current study has been designed to expand on that methodology to determine and quantify age-related changes in the size of the TM and TM interface shadow.

## 2. Methods

This prospective cohort study was conducted at the Robert Cizik Eye Clinic of the Ruiz Department of Ophthalmology and Visual Science at The University of Texas Medical School, Houston, TX. Institutional Review Board approval was obtained from The University of Texas Health Science Center Committee for the Protection of Human Subjects. All research adhered to the tenets of the Declaration of Helsinki and was HIPAA compliant.

### 2.1. Participants

Patients, their family members, and/or staff 18 years of age or older were recruited from the Robert Cizik Eye Clinic. After explaining the nature and possible consequences of the study, informed consent was obtained from each participant. After obtaining informed consent, demographic data were recorded. Participants underwent slit lamp examination, intraocular pressure (IOP) measurement, and gonioscopic examination performed by one of the glaucoma specialists (RMF or NPB) in a dark room using a Posner goniolens without compression. Eyes with open angles (Spaeth score *C*, *D*, or *E* [14]) were selected. Subjects over the age of 80 and who used any medication that may likely have affected angle anatomy at the time of imaging or within the past month were excluded. Eyes with IOP greater than 21 mm Hg were excluded. Eyes with any previous intraocular surgery or any anterior segment abnormality that affected the angle or its measurements (i.e., significant corneal opacity) were also excluded. Fifteen participants from each of three age groups (18–40, 41–60, and 61–80) met eligibility criteria and were enrolled. When both eyes of the participant were eligible, one eye was randomly selected.

### 2.2. ASOCT Imaging

The CASIA SS-1000 Fourier domain (FD-) ASOCT (Tomey Corporation, Nagoya, Japan) is a swept-source FD-ASOCT that uses 1,310 nm wavelength light with a scan speed of 30,000 A-scans per second to image the anterior chamber, including the angle recess. Images can be obtained in high resolution 2D mode (2048 A-scans each, 1 pixel = 7.9 *μ*m × 10.0 *μ*m). Both horizontal and vertical plane scans are completed simultaneously in 0.2 seconds. All radial scans are 16 mm in length and 6 mm in depth [15].

### 2.3. Acquisition of ASOCT Images

All eyes were imaged in a dark room by 2 operators. One held the participant's eyelids open while the other operated the ASOCT. To keep the participant's eyelids open, both index fingers were placed at the eyelid margins, and the eyelids were separated to allow visualization of the superior and inferior limbus. Pressure from the index fingers was directed to the superior and inferior orbital rims to avoid pressure on the globe. Participants were instructed to focus on the internal fixation light. After adjusting the participant's position, eyes were scanned in 2D mode using the anterior segment scan type and the autoalignment function. Using the autoalignment function allows for reliable fixation. 

### 2.4. Analysis of ASOCT Images

**Figure 1:**
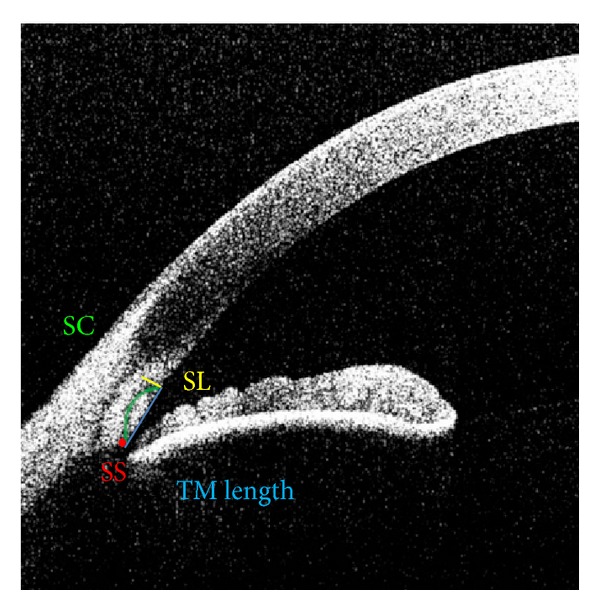
Anterior chamber angle anatomy. Anterior segment optical coherence tomography (ASOCT) image exhibiting Schlemm's canal (SC, green line), Schwalbe's line (SL, yellow line), and the scleral spur (SS, red dot). Trabecular meshwork (TM) length as measured is illustrated by the blue line.

Several anatomic structures were defined as follows (Figure 1):

- scleral spur (SS)—the point where there was a change in curvature in the corneoscleral-aqueous interface, often appearing as an inward protrusion of the sclera [9];
- Schwalbe's line (SL)—the point where the anterior end of TM meets the peripheral end of the corneal endothelium;
- Schlemm's canal (SC)—tubular canal located at the sclerocorneal junction.

The TM was bordered by the SS, the posterior endpoint of the SC, and SL. TM length was defined as the length between SS and SL [13]. Previous studies have shown that FD-ASOCT is capable of imaging TM and SC [8, 10, 11], and the measurements made are very repeatable [13]. However, a hyporeflective band wrapping around the TM, defined by Kagemann et al. as the TM interface shadow, has the potential to be improperly included in the measurement of TM. These TM interface shadows often completely surround the TM on the anterior, lateral, and even posterior sides, erroneously increasing the TM measurements if not recognized and separated [11]. 

**Figure 2:**
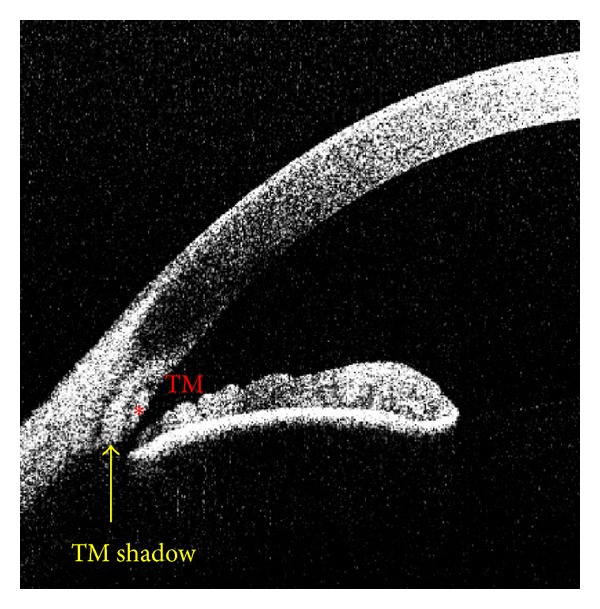
Anterior chamber angle anatomy. Anterior segment optical coherence tomography (ASOCT) image with trabecular meshwork (red asterisk) and trabecular meshwork interface shadow (yellow arrow) labeled.

**Figure 3:**
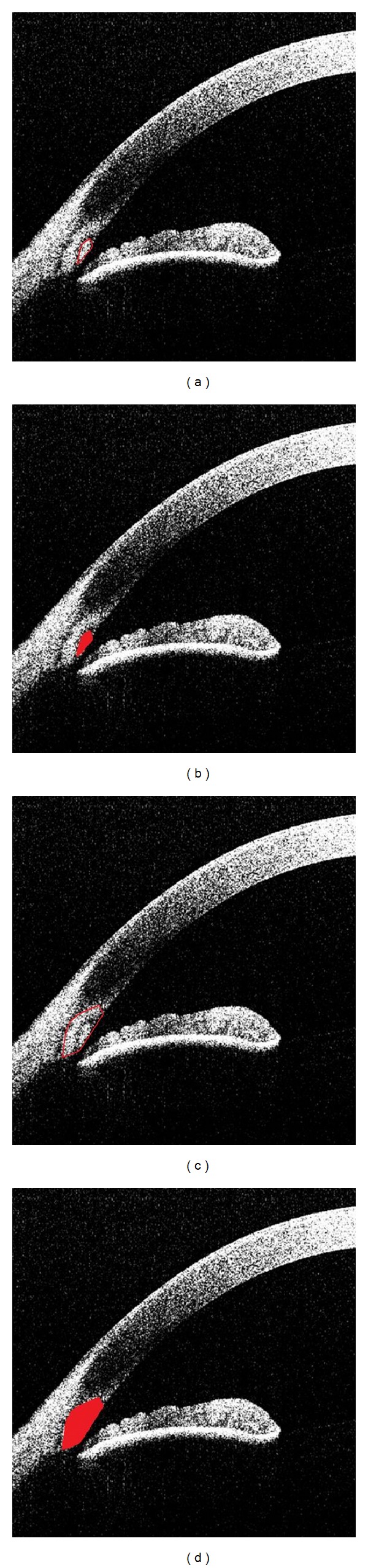
Anterior chamber angle parameters as identified using ACAI software. (a) Outline of the trabecular meshwork (TM) area. (b) Highlighted TM area. (c) Outline of TM interface shadow area. (d) Highlighted TM interface shadow area.

Two readers (MEG and SK) were trained by known expert observers (glaucoma specialists, NPB and RMF) to identify SC and SL using images from patients who underwent canaloplasty (nonstudy patients), because Schlemm's canal and hence TM are made apparent by the presence of suture material. After training, the readers independently graded visibility of SL and SC (0 = not visible; 1 = visible; see Supplementary Figure 1 available online at http://dx.doi.org/10.1155/2013/295204) for all 4 angles (nasal, temporal, superior, and inferior) and measured the area and the length of TM as well as the TM interface shadow at the temporal angles using proprietary software (ACAI, Houston, Texas) (Figures 2 and 3).

### 2.5. Statistical Analysis

Demographics were summarized by mean and standard deviation (SD) for continuous variables or by frequency (%) for discrete variables. Interobserver reproducibility was evaluated using an intraclass correlation coefficient (ICC) in a random intercept model. An ICC ≤ 0.4 was defined as poor reproducibility, between 0.4 and 0.70 was defined as fair to good reproducibility, and ≥0.70 was defined as excellent reproducibility [16]. To evaluate the effect of age (as a continuous variable) on TM, the average of each TM parameter was obtained from 2 readers and adjusted for sex (male versus female), race (White versus non-White), and IOP. Gonioscopy scores (*E* versus *C* and *D*) were evaluated using stepwise regression analysis.

Usui et al. reported that mean TM area was approximately 0.065 (±0.006) mm^2^ using an HD scan mode [13]. The variation was anticipated to be higher in a lower resolution scan mode. A sample size of 45 is sufficient to detect a minimum of 5% reduction of TM area (*≈*0.00325 mm^2^) per decade of age at 5% significance level and 80% power, assuming standard deviation of age and TM area was (80 − 20)/4 = 15 years and 0.012 mm^2^, respectively.

All statistical analyses were performed using SAS for Windows v9.2 (SAS Institute, Inc., Cary, NC). *P* < 0.05 was considered statistically significant.

## 3. Results

Of the 45 participants, 28 (62%) were female; the mean age was 49.0 (±16.4) years, and the mean IOP was 16.4 (±2.5) mm Hg. The study included 29 White (64.4%), 8 Hispanic (17.8%), 6 Black (13.3%), and 2 Asian (4.4%) participants. Gonioscopic findings included 27 eyes (60%) open to the ciliary body band (*E*), 13 eyes (28.9%) open to the scleral spur (*D*), and 5 eyes (11%) open only to the posterior TM (*C*). All images were reviewed by 2 independent readers for SC and SL visibility in each angle. There were 25 (55.6%) SCs and 30 (66.7%) SLs visible by both readers in nasal quadrants, 41 (91.1%) SCs and 44 (97.8%) SLs visible in temporal quadrants, 28 (48.9%) SCs and 25 (55.6%) SLs visible in superior quadrants, and 28 (62.2%) SCs and 31 (68.9%) SLs visible in inferior quadrants. The temporal quadrant had the best visibility, allowing readers to identify the landmarks and measure the TM and TM interface shadow. Thus, only the statistical analyses of the temporal TM measurements were included.

Table 1 summarizes the means (±SD) of TM parameters identified by each reader and their differences and ICCs. The mean difference in TM area was −0.0005 (±0.0058) mm^2^ and in TM length was 0.0067 (±0.1290) mm. Similarly, the mean difference in TM interface shadow area was 0.0123 (±0.0285) mm^2^ and in TM interface shadow length was 0.0043 (±0.1233) mm. The ICCs ranged from 0.45 (in TM length) to 0.82 (in TM area). The results indicated that ICCs were good (0.40–0.70) or excellent (>0.70) [16]. 

The average TM parameters by 2 readers were 0.0487 (±0.0092) mm^2^ for TM area, 0.5502 (±0.1033) mm for TM length, 0.1623 (±0.0341) mm^2^ for TM interface shadow area, and 0.7755 (±0.1574) mm for TM interface shadow length. The TM interface shadow area is about 3 times that of the TM area while the TM interface shadow length is about 40% longer than the TM length.

Stepwise regression analysis showed that after adjusting for gonioscopy score (*E* versus *C* and *D*), the TM interface shadow area increased 0.0087 (±0.0030, *P* = 0.0052) mm^2^, which was about 5% of the average size, with every decade of age. The gonioscopy score was a factor affecting TM length (*P* = 0.0167) while age was not (*P* = 0.0532). Those eyes with a gonioscopy score of *E* (ciliary body band) were 0.0740 (±0.0297) mm longer in TM length than those with a gonioscopy score *C* or *D*. However, the TM area and TM interface shadow length were not correlated with either age or gonioscopy score. Race, sex, and IOP were not correlated with any of the TM parameters.

## 4. Discussion

The trabecular meshwork (TM) is a collagenous tissue bordered by scleral spur (SS), the posterior edge of Schlemm's canal (SC), and Schwalbe's line (SL) [8]. The birefringent nature of the TM creates a diffuse hyperscattering region in an ASOCT image, known as the TM interface shadow (Figures 2 and 3). This shadow has also been visualized using polarization ASOCT [8]. Unfortunately, most previous publications on ASOCT looking at the TM have not explicitly identified or evaluated the TM shadow. While the TM area and length provide information for gross anatomic structures, the TM interface shadow may reflect alterations at the microscopic/cellular level. Well-documented examples of OCT changes reflecting cellular alterations (i.e., macular thickness and pathology) have been reported in retinal imaging [17, 18].

This prospective observational study demonstrated that on ASOCT images, the TM is more visible in the temporal quadrants (>90%) compared to the superior quadrants (<30%). This study also demonstrated that the TM interface shadow area increases with age. Moreover, participants with angles open to the ciliary body band had longer TM lengths than those with narrower angles (not open to the ciliary body band).

This study found that the TM could be identified more clearly in the temporal quadrants compared to other quadrants, which was similar to the findings of Yasuno et al. [8]. Potential factors related to poorer identification in other quadrants may include the angle of image acquisition, eyelid/lash artifacts, and potentially quadratic differences in ocular anatomy. The TM length and area found in this study are similar to the values measured by Usui et al. [13]. Neither the measurements for TM length in this study nor those of Usui et al. were similar to the findings of Tun et al. [19] and Day et al. [20]. The TM length values found by Tun et al. and Day et al. are very similar to the values found in this study for the length of the TM interface shadow, indicating that they may have included the shadow in their analysis of TM size. 

Although our study found no significant correlation between the actual TM and age, there was a significant difference in the area of the TM interface shadow with age in open angle eyes. The TM interface shadow may be the result of reflection of light off TM, which is dependent on TM density. Decreased elasticity of the TM is observed with age [21]. Thickening of the TM elastic fibers (leading to formation of extracellular “sheath-derived plaques”) increases stability but decreases the drainage of aqueous humor through pores of the cribriform net. Increased extracellular matrix leads to increased resistance to flow. With each decade of age, an increase in TM density causes increased light reflection and scattering, explaining our findings of a 5% increase per decade of age in the TM interface shadow area. This should be explored to determine a potential relationship between the TM interface shadow and trabecular outflow resistance.

The TM interface shadow area is 3 times larger and the length is 40% longer than the TM area and length, which may be explained by amplification of the reflected signals. Light shining on the TM is reflected and scattered, causing a TM interface shadow. The closer an object is to the light source, the more the light is blocked out and the larger the shadow is. Also, the angle of incoming/reflected light during image acquisition impacts the shadow area and size, a variable which was limited by consistent study imaging technique. Additionally, as the distance between the object blocking light and the surface of projection increases, the shadow area/length proportionally increases. The distance of the TM (the object blocking the light) from the signal detector (surface of projection) may have contributed to the proportional increase in the TM interface shadow (the shadow). Consequently, an increase in the TM interface shadow area can potentially be correlated to a proportional increase in the TM area.

There are several limitations in the present study. The current imaging technique cannot achieve the required visibility in nasal, superior, and inferior quadrants as it can in the temporal quadrant. Better images could be obtained using the ASOCT Angle HD mode for each angle; however, the lack of reproducible fixation in this mode limits image (and therefore data) reproducibility. Second, automated measurements of the TM and TM interface shadow parameters could eliminate the possibility of manually introduced errors. Due to a lack of software that could automatically detect the boundaries of TM and TM interface shadow, free-hand techniques were employed using trained readers (see Section 2). Third, besides the TM area and length, other parameters need to be investigated, such as the relationship between the TM interface shadow intensity and area and the change in intensity of the TM in order to study the theory of plaque buildup over time. Fourth, refractive error and axial length were not measured, which could help explain whether myopia is associated with deeper angles and if it can be translated to longer TM length. Additionally, only using temporal images for analysis may have confounded the results, but it would be expected that age-related changes would occur diffusely. Further studies are necessary to better understand the *in vivo* implications associated with the age-related structural changes in the TM.

Changes associated with aging (either natural or disease related) may be more prevalent in older participants and may play an unknown role in anatomy of the TM. This study attempted to exclude any potential participants with abnormal ocular anatomy. However, one can never be certain of subclinical abnormalities. This is a limitation that may have biased the study in an unknown way. Also, limitations in reproducibility may have prevented the detection of an age-related difference where one may have existed

In conclusion, this study measures TM *in vivo* in humans. It is the first study to determine the relationship between the anatomic length/area of the TM interface shadow and age. This study demonstrated that there are *in vivo* anatomic changes associated with age that have not been previously described. 

## Supplementary Material

Supplementary Figure 1: Examples of anterior segment optical coherence tomography (ASOCT) scans in all quadrants labeled where Schlemm's canal (SC; red arrow) and Schwalbe's line (SL; yellow arrow) were determined to be not visible (graded as 0) and visible (graded as 1) and the approximate locations of each structure. Temporal quadrant images were only used for data analysis in this study because they had the most consistent angle visibility (see Methods).
Supplementary Figure 2: Example of anterior segment optical coherence tomography (ASOCT) scan where the trabecular meshwork (TM) and TM interface shadow were not visible.

## Figures and Tables

**Table 1:** Means ± SD of trabecular meshwork (TM) parameters identified by each reader and their differences and interobserver correlation coefficients (ICC).

TM^a^ parameter	Reader MG	Reader SR	Difference	ICC^b^
TM area (mm^2^)	0.0484 ± 0.0097	0.0490 ± 0.0096	−0.0005 ± 0.0058	0.82
TM length (mm)	0.5536 ± 0.1147	0.5468 ± 0.1284	0.0067 ± 0.1290	0.45
TM interface shadow area (mm^2^)	0.1684 ± 0.0374	0.1561 ± 0.0365	0.0123 ± 0.0285	0.66
TM interface shadow length (mm)	0.7776 ± 0.1516	0.7733 ± 0.1848	0.0043 ± 0.1233	0.74

^a^TM: trabecular meshwork; ^b^ICC: interobserver correlation coefficient.
