# Supplementary figures and images for: Age-Related Changes in Trabecular Meshwork Imaging
Source: Biomed Res Int. 2013 Sep 19;2013:295204. doi: 10.1155/2013/295204 (PMC3791583; doi:10.1155/2013/295204)

## Slide 1
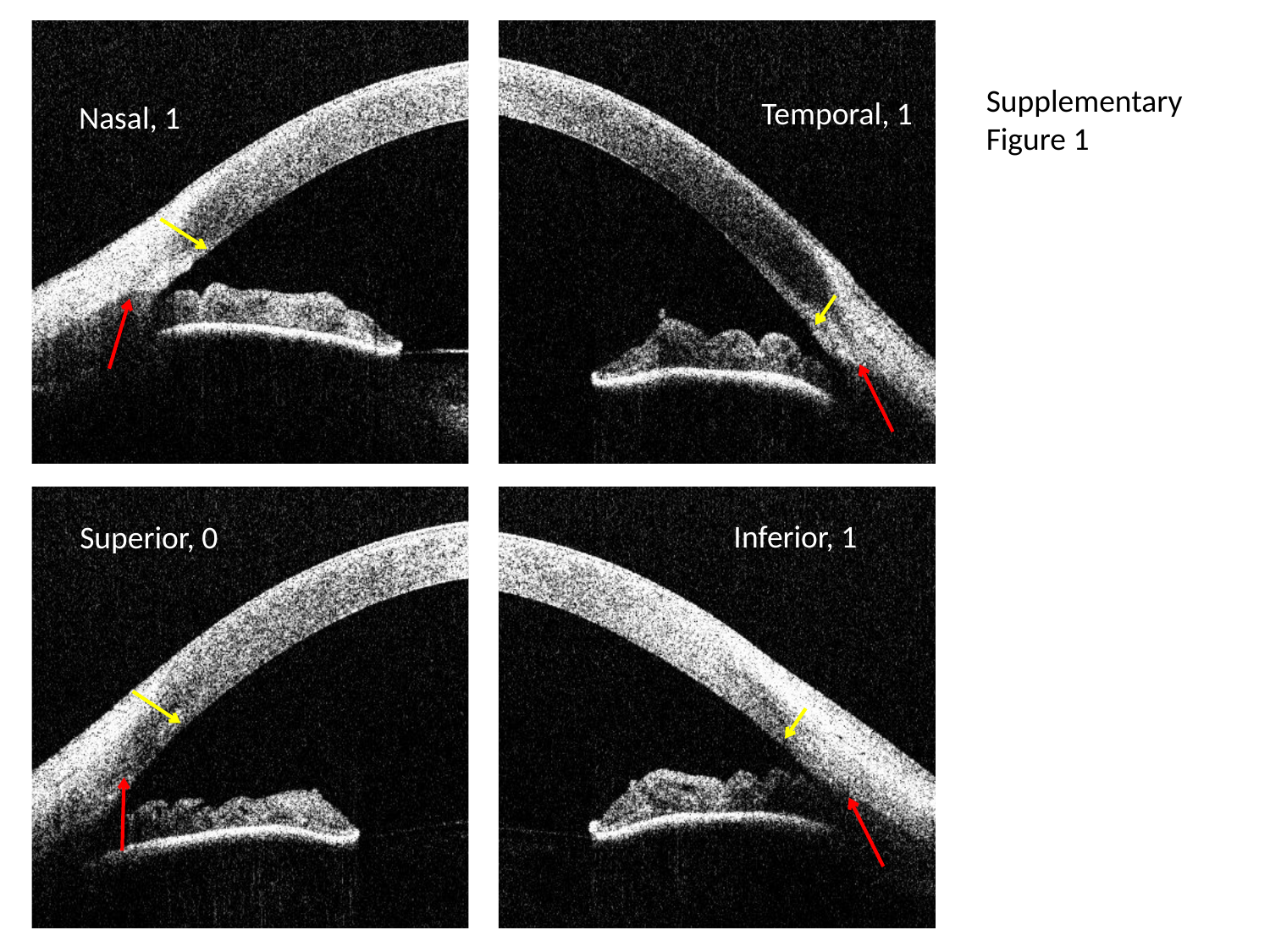

Nasal, 1
Temporal, 1
Superior, 0
Inferior, 1
Supplementary
Figure 1

## Slide 2
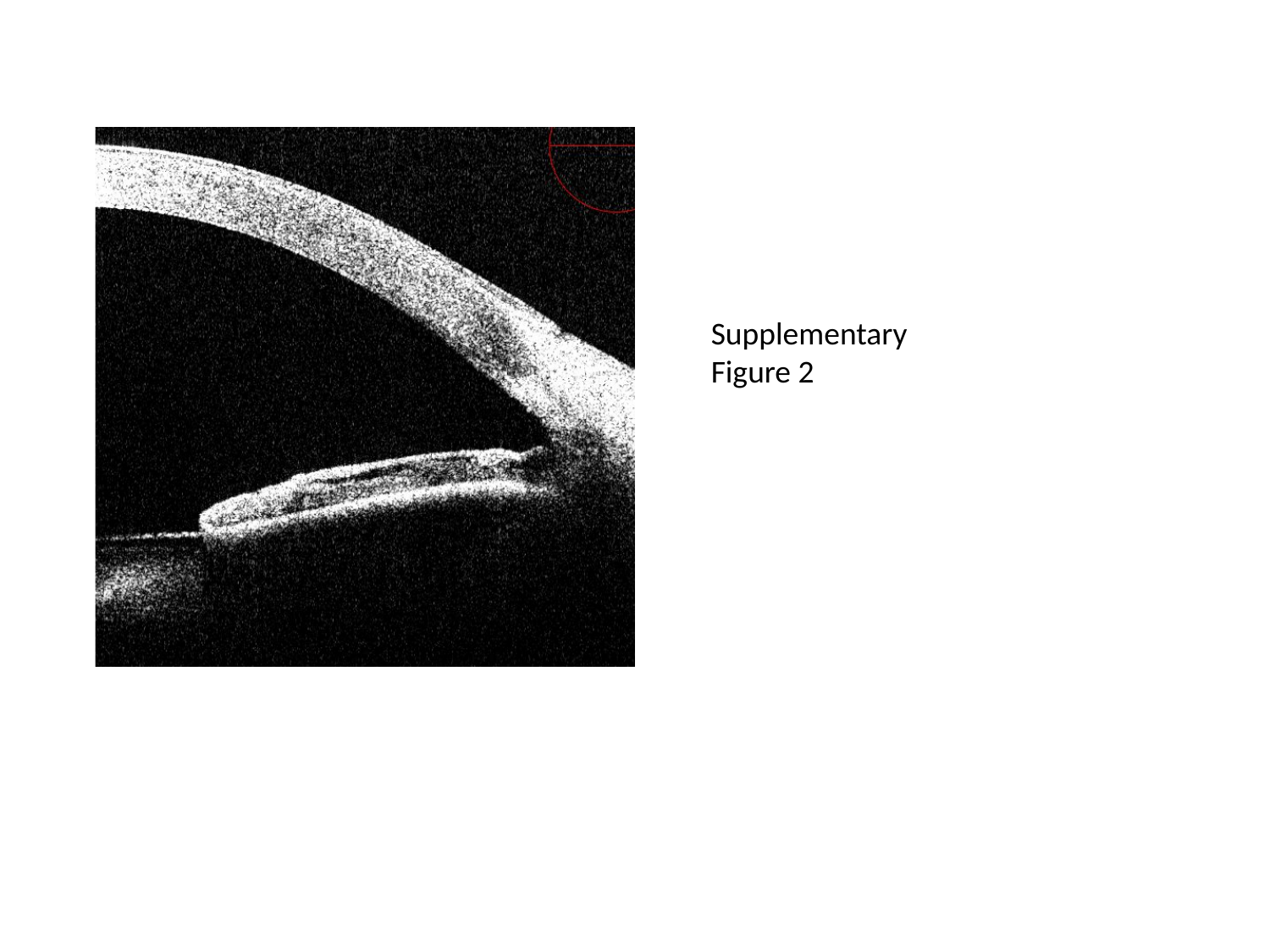

Supplementary
Figure 2

Supplement: Supplementary file 1 — Supplementary Figure 1: Examples of anterior segment optical coherence tomography (ASOCT) scans in all quadrants labeled where Schlemm's canal (SC; red arrow) and Schwalbe's line (SL; yellow arrow) were determined to be not visible (graded as 0) and visible (graded as 1) and the approximate locations of each structure. Temporal quadrant images were only used for data analysis in this study because they had the most consistent angle visibility (see Methods). Supplementary Figure 2: Example of anterior segment optical coherence tomography (ASOCT) scan where the trabecular meshwork (TM) and TM interface shadow were not visible. [file 295204.f1.pptx]
